# Supplementary material for: Deciphering Differences in Microbial Community Diversity between Clubroot-Diseased and Healthy Soils
Source: Microorganisms. 2024 Jan 25;12(2):251. doi: 10.3390/microorganisms12020251 (PMC10893227; doi:10.3390/microorganisms12020251)
Supplement: Supplementary file 1 [file microorganisms-12-00251-s001.zip › microorganisms-2819078-supplementary.pdf]

Supplementary materials

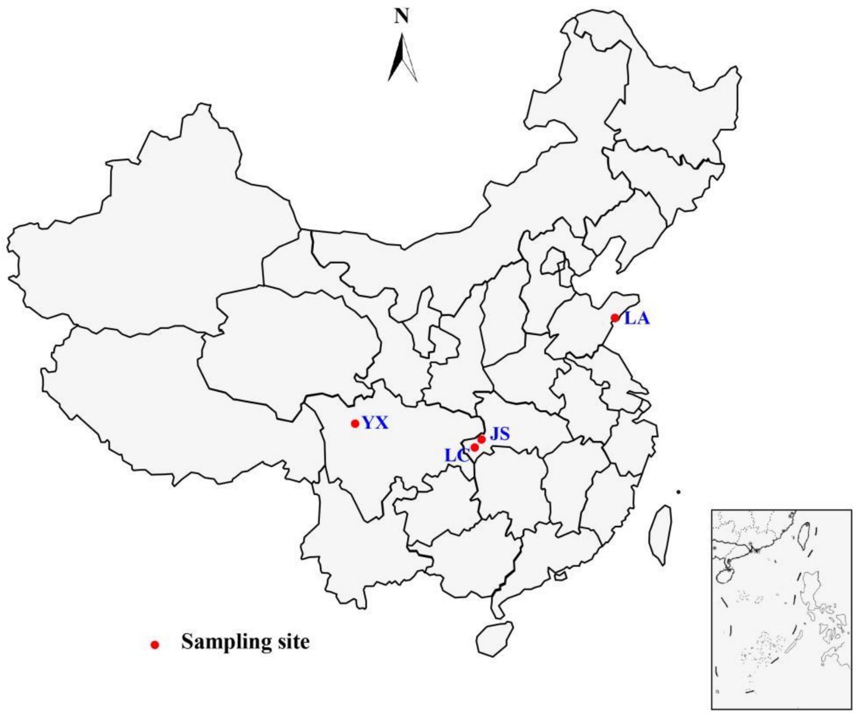

Figure S1 Sampling sites. For each field, 3 random subplots (approximately 60 m^2) were chosen, and soil samples from approximately 10 healthy (healthy soil) or 10 clubroot-diseased (diseased soil) plants from each subplot were collected using the checkerboard sampling method during August 2020. Briefly, each subplot was divided into 10 areas, and rhizosphere soils of healthy and clubroot-diseased plants in the central point of each area were collected by manual shaking. In each field, not only did the healthy plants have no root gall, but the aboveground plant growth was also good and consistent. Accordingly, the diseased plants showed noticeable wilting in the aboveground parts, and the root systems showed typical gall formation. The 10 healthy or 10 diseased soils were mixed to form one composite sample. The composite samples were placed into separate sterile bags and transported to the laboratory on ice. LA, Licang, Shandong Province; YX, Youxian, Sichuan Province; LC, Lichuan, Hubei Province; JS, Jianshi, Hubei Province.

Table S1 The keystone taxa identified as connectors in the soil fungal networks.

Role	Phylum	Genus	Pi	Zi
Connectors	Ascomycota	*Acremonium*	0.750	1.155
Connectors	Ascomycota	*Cephalotrichum*	0.679	0.694
Connectors	Ascomycota	*Clonostachys*	0.750	-1.095
Connectors	Ascomycota	*Cosmospora*	0.750	-0.953
Connectors	Ascomycota	*Cyphellophora*	0.750	-0.953
Connectors	Ascomycota	*Dactylonectria*	0.750	-0.694
Connectors	Ascomycota	*Fusarium*	0.778	-1.076
Connectors	Ascomycota	*Lasiosphaeris*	0.750	-0.685
Connectors	Ascomycota	*Lecythophora*	0.778	0.730
Connectors	Ascomycota	*Microdochium*	0.750	0.000
Connectors	Ascomycota	*Monocillium*	0.688	0.730
Connectors	Ascomycota	*Myrothecium*	0.750	0.730
Connectors	Ascomycota	*Pleurostoma*	0.750	-0.577
Connectors	Ascomycota	*Preussia*	0.750	-0.953
Connectors	Basidiomycota	*Amylostereum*	0.750	-0.694
Connectors	Basidiomycota	*Clitopilus*	0.750	-0.685
Connectors	Basidiomycota	*Dermocybe*	0.750	0.000
Connectors	Basidiomycota	*Hannaella*	0.750	-0.694
Connectors	Basidiomycota	*Leucocoprinus*	0.750	1.155
Connectors	Basidiomycota	*Myriococcum*	0.750	-0.953
Connectors	Chytridiomycota	*Clydaea*	0.688	-0.114
Connectors	Glomeromycota	*Paraglomus*	0.750	1.155

Table S2 The keystone taxa identified as connectors in the soil bacterial networks.

Role	Phylum	Genus	Pi	Zi
Connectors	Proteobacteria	*Acidovorax*	0.778	0.378
Connectors	Proteobacteria	*Alcaligenes*	0.750	0.866
Connectors	Proteobacteria	*Arenimonas*	0.750	0.000
Connectors	Proteobacteria	*Caenimonas*	0.750	0.000
Connectors	Proteobacteria	*Ellin6067*	0.750	-1.413
Connectors	Proteobacteria	*Klebsiella*	0.778	0.378
Connectors	Proteobacteria	*Mitsuaria*	0.750	0.000
Connectors	Proteobacteria	*Nitrosospira*	0.630	-1.029
Connectors	Proteobacteria	*Pectobacterium*	0.750	0.000
Connectors	Proteobacteria	*Polycyclovorans*	0.750	-1.505
Connectors	Proteobacteria	*Pseudochrobactrum*	0.750	-1.413
Connectors	Proteobacteria	*Pseudoxanthomonas*	0.750	-1.413
Connectors	Proteobacteria	*Rhodanobacter*	0.688	0.378
Connectors	Proteobacteria	*Uliginosibacterium*	0.778	0.730
Connectors	Proteobacteria	*Vogesella*	0.750	0.000
Connectors	Acidobacteriota	*Candidatus_Koribacter*	0.750	1.155
Connectors	Actinobacteriota	*Actinoplanes*	0.750	-1.505
Connectors	Actinobacteriota	*Glycomyces*	0.750	1.155
Connectors	Actinobacteriota	*Nocardia*	0.750	-1.505
Connectors	Actinobacteriota	*Phycicoccus*	0.633	-0.389
Connectors	Bacteroidota	*Myroides*	0.750	0.866
Connectors	Bacteroidota	*Terrimonas*	0.688	1.701
Connectors	Chloroflexi	uncultured_Chloroflexi_bacterium	0.630	-1.029
Connectors	Myxococcota	*Pajaroellobacter*	0.688	0.730
Connectors	Nitrospirota	*Nitrospira*	0.750	0.000
Connectors	Verrucomicrobiota	*Chthoniobacter*	0.750	1.155
